# Manifold learning and maximum likelihood estimation for hyperbolic network embedding

**DOI:** 10.1007/s41109-016-0013-0

**Published:** 2016-11-15

**Authors:** Gregorio Alanis-Lobato, Pablo Mier, Miguel A. Andrade-Navarro

**Affiliations:** 1 Institute of Molecular Biology, Ackermannweg 4, Mainz, 55128 Germany; 2 Faculty of Biology, Johannes Gutenberg Universität, Gresemundweg 2, Mainz, 55128 Germany

**Keywords:** Complex networks, Hyperbolic geometry, Manifold learning, Maximum likelihood estimation, Network embedding, Network geometry, Graph Laplacian

## Abstract

The Popularity-Similarity (PS) model sustains that clustering and hierarchy, properties common to most networks representing complex systems, are the result of an optimisation process in which nodes seek to form ties, not only with the most connected (popular) system components, but also with those that are similar to them. This model has a geometric interpretation in hyperbolic space, where distances between nodes abstract popularity-similarity trade-offs and the formation of scale-free and strongly clustered networks can be accurately described.

Current methods for mapping networks to hyperbolic space are based on maximum likelihood estimations or manifold learning. The former approach is very accurate but slow; the latter improves efficiency at the cost of accuracy. Here, we analyse the strengths and limitations of both strategies and assess the advantages of combining them to efficiently embed big networks, allowing for their examination from a geometric perspective. Our evaluations in artificial and real networks support the idea that hyperbolic distance constraints play a significant role in the formation of edges between nodes. This means that challenging problems in network science, like link prediction or community detection, could be more easily addressed under this geometric framework.

**Electronic supplementary material:**

The online version of this article (doi:10.1007/s41109-016-0013-0) contains supplementary material, which is available to authorized users.

## Introduction

The network representation of many complex systems, like the Internet or the protein interactome, shows characteristics commonly present in geometric objects; scale invariance and self-similarity amongst them (Barabási and Albert 1999; Song et al. 2006; Goh et al. 2006; Serrano et al. 2008). It is then no surprise that several models, aimed at mimicking the evolution and formation of these networks, assume the existence of a hidden geometry underlying their structure and shaping their topology (Aste et al. 2005; Aste et al. 2012; Boguñá et al. 2009; Dall and Christensen 2002; Ferretti and Cortelezzi 2011; Krioukov et al. 2010; Papadopoulos et al. 2012; Serrano et al. 2008) (we refer the reader to (Barthélemy 2011) for an extensive review on the subject).

Of special interest is the so-called Popularity-Similarity (PS) model, which sustains that strong clustering and scale-free node degree distributions are the result of an optimisation process involving two measures of attractiveness: node popularity and similarity between nodes (Papadopoulos et al. 2012). On the one hand, popularity reflects the ability of a node to attract connections from other nodes over time, and it is thus associated with its seniority status in the system. On the other, nodes that are similar simply tend to connect, regardless of their rank.

The PS model has a geometric interpretation in hyperbolic space, where the trade-offs that new nodes have to optimise when joining a system are abstracted by the hyperbolic distance between them and existing ones (Krioukov et al. 2010; Papadopoulos et al. 2012). In this model, a network lies within a hyperbolic disc of radius *R*∼ ln*N*, where *N* is the total number of nodes. The popularity dimension is represented by radial node coordinates *r*_*i*_, with senior nodes in close proximity to the disc’s centre. The similarity dimension is associated with the angular positioning of nodes *θ*_*i*_ and short hyperbolic distances between them (approximately *x*_*ij*_=*r*_*i*_+*r*_*j*_+2 ln(*θ*_*ij*_/2) for any two nodes *i* and *j* separated by an angle *θ*_*ij*_) correspond to high probabilities of link formation.

With the simplest version of the model, we can produce networks with scaling exponent *γ*=2 in the so-called cold-temperature regime (*T*=0), where their clustering is the strongest possible (Krioukov et al. 2010; Papadopoulos et al. 2012). To increase the value of *γ*, we can simulate popularity fading by moving senior nodes away from the disc’s centre. To decrease clustering, networks can be submitted to higher temperatures (*T*>0, see Methods). These additional mechanisms give place to a very versatile model to study network dynamics (Papadopoulos et al. 2012).

If the PS model can generate networks that are similar to those we observe in nature and engineering (Krioukov et al. 2010; Papadopoulos et al. 2012), does it mean that packets travelling the Internet, signals going from receptors to transcription factors in the cell or messages between people in social networks traverse the hyperbolic geometry underlying each of these systems? To answer this question, we need a means to map them to hyperbolic space, to then check whether hyperbolically close nodes tend to connect more than distant ones, and assess whether information travels efficiently through the network topology.

**Fig. 1:**
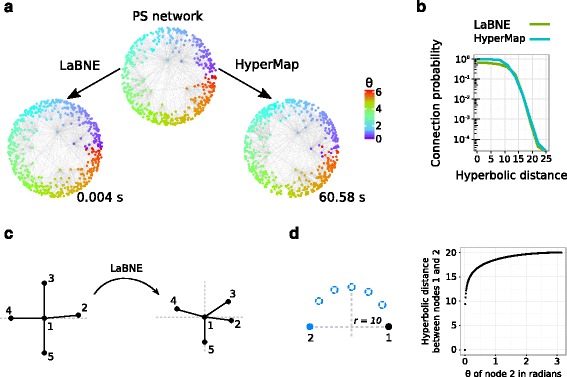
Issues with hyperbolic embeddings by LaBNE. **a** An artificial network produced by the PS model, with 500 nodes, 2*m*=10, *γ*=2.5 and *T*=0.3, is mapped to hyperbolic space with LaBNE and HyperMap. Even when LaBNE’s embedding is extremely fast and its inferred node positions, indicated with colours, coincide with those from the input network, panel **b** shows that HyperMap is more accurate, because the probability of finding connected nodes at small hyperbolic distances is higher than if LaBNE’s coordinates are used. We illustrate the reasons for this with the simple 5-node network of panel **c** and its embedding by LaBNE. Despite the fact that the inferred and real angular coordinates, together with the corresponding hyperbolic distances, are highly correlated (Pearson correlations of 0.97 and 0.98, respectively), it is clear that the angular positions of nodes 2, 3 and 4 are smaller than they should be. This is a consequence of LaBNE’s aim to map connected nodes as close as possible in the embedding space, disregarding that disconnected nodes should be far from each other. Although this does not have a big impact in Euclidean embeddings, it can be very problematic in hyperbolic space. Panel **d** shows how the hyperbolic distance between nodes 1 and 2 changes dramatically, even for small changes in the angular coordinate of the latter

In 2015, Papadopoulos and colleagues introduced HyperMap, a Maximum Likelihood Estimation (MLE) approach, in which the space of PS models with the same structural properties as the network of interest is explored, in search for the one that better fits its topology (see Methods and (Papadopoulos F et al. 2015b; Papadopoulos et al. 2015a) for more details). This search is very accurate, albeit computationally demanding (see Fig. 1 a, b), which means that HyperMap requires of correction steps or heuristics in order to make it suitable for big networks (Papadopoulos et al. 2015a).

Inspired by the well-established field of non-linear dimensionality reduction in Machine Learning (Cayton 2005), we recently put forward the Laplacian-based Network Embedding or LaBNE (Alanis-Lobato et al. 2016). In manifold learning, most algorithms rely on the construction of a mesh or network connecting nearby samples contained in a high-dimensional manifold (Cayton 2005; Zemel and Carreira-Perpiñán 2004). If there is really a hyperbolic geometry underlying a complex network, it should lie on a hyperbolic plane, with nodes drifting away from the space origin. Thus, the network itself can be seen as the mesh that connects samples (nodes in this case) that are close to each other (Papadopoulos et al. 2012) and serve as the basis to recover the hyperbolic coordinates of its nodes (see Methods and (Alanis-Lobato et al. 2016) for more details). LaBNE is extremely fast (see Fig. 1 a), but highly depends on topological information to carry out good embeddings. This means that the higher the average node degree (2*m*) and clustering coefficient ($\bar {c}$) of a network, the better the results it achieves (Alanis-Lobato et al. 2016). In addition, LaBNE’s aim to map connected nodes as close as possible to each other in the embedding space, disregarding that disconnected nodes should be far apart (Shaw and Jebara 2009), can lead to inaccuracies when associating short hyperbolic distances with connections between nodes (see Fig. 1 b-d).

In the present article, we assess the pros and cons of both strategies and introduce a hybrid approach that pursues a more efficient and accurate network embedding into the two-dimensional hyperbolic plane $\mathbb {H}^{2}$, represented by the interior of a Euclidean circle (Krioukov et al. 2010). We carry out analyses on artificial and real networks and, based on the results, discuss the strengths and limitations of these hyperbolic mapping techniques.

## Results

### LaBNE+HM and its performance in artificial networks

Given the drawbacks and limitations of both HyperMap and LaBNE, we aimed at combining them to improve LaBNE’s accuracy and reduce HyperMap’s execution times. We focused on undirected, unweighted, single-component networks and assumed that they are scale-free (with scaling exponent *γ*∈[ 2,3]) and have a clustering coefficient $\bar {c}$ significantly larger than expected by chance.

**Fig. 2:**
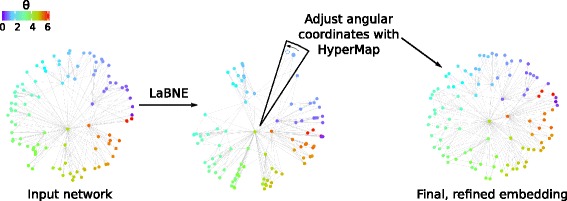
LaBNE+HM. An undirected, unweighted, single-component network with scale-free node degree distribution and strong clustering can be input to LaBNE+HM to reveal the hyperbolic geometry underlying it. A first hyperbolic arrangement of nodes is obtained by LaBNE (a manifold learning approach), which is later refined by HyperMap (a maximum likelihood estimation approach). In LaBNE+HM, the space of possible PS models that HyperMap has to explore is greatly reduced, as it only needs to search for appropriate node angular coordinates in a small window around the angles found by LaBNE. In the schematic, the angular coordinate of a node is refined by increasing its value

LaBNE+HyperMap (LaBNE+HM), our proposed approach, uses LaBNE to quickly draft a first geometric configuration of a network of interest in $\mathbb {H}^{2}$. This draft is then passed on to HyperMap, which refines the embedding and produces the final mapping to hyperbolic space. LaBNE+HM profits from LaBNE’s fast embeddings and significantly reduces the search space of HyperMap, which instead of trying to find the best angular coordinate of each node in the range [ 0,2*π*], now only needs to focus on a window around the angles found by LaBNE (see Fig. 2). The size of this window depends on the quality of LaBNE’s embeddings, which, as above-mentioned, are better in networks with high 2*m* and $\bar {c}$ (low *T*). This means that the window should be narrow in strongly clustered networks, but wider if they are sparser. This has an impact on LaBNE+HM’s execution time, which should be expected to behave as LaBNE if the window is close to 0 and as HyperMap if it is close to 2*π*. It is also important to mention that, due to rotational invariance of distances, the set of hyperbolic coordinates responsible for the edges observed in a network is not unique (Alanis-Lobato et al. 2016). Therefore, the goal of the proposed technique is not to find a specific set of coordinates, but the one that corresponds better with the hyperbolic, distance-dependent connection probabilities that produce the network of interest.

To investigate the performance of LaBNE, HyperMap and LaBNE+HM in a controlled manner, we generated artificial networks with different structural characteristics using the PS model (*N*=500, 2*m*=10, *γ*={2.25,2.5,2.75} and *T*={0,0.3,0.6,0.9}). Based on the above-mentioned rationale regarding LaBNE+HM’s required window for different temperatures, we used windows *w*=*π*/36 radians (5°) for *T*=0, *w*=*π*/6 radians (30°) for *T*=0.3, *w*=*π*/4 radians (45°) for *T*=0.6 and *w*=*π*/3 radians (60°) for *T*=0.9 in our analyses. For each node *i*, different angles separated by 1/*i* radians are considered within such windows (here *i*={1,2,…,*N*} is the rank of each node, when they are sorted decreasingly by degree).

**Fig. 3:**
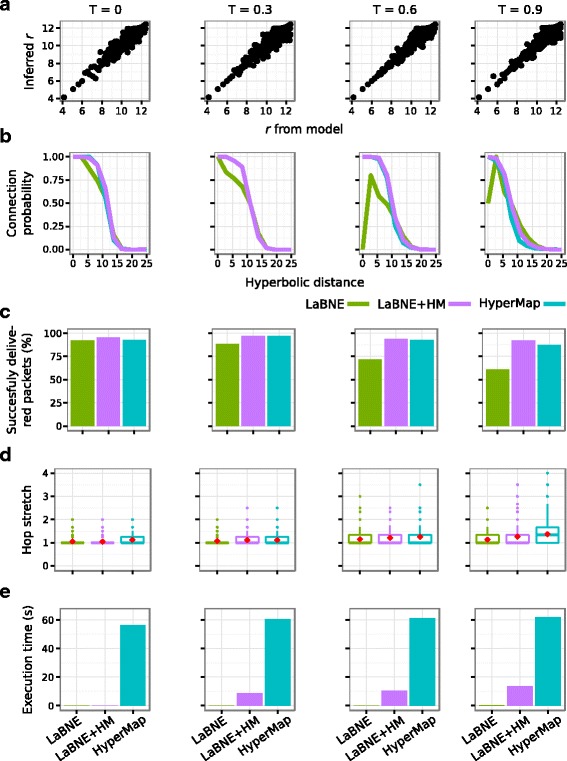
Benchmarking on artificial networks (*γ*=2.5). Artificial networks with *N*=500 nodes, average node degree 2*m*=10, scaling exponent *γ*=2.5 and temperature *T*={0,0.3,0.6,0.9} were embedded to hyperbolic space with LaBNE, HyperMap and LaBNE+HM. **a** Real vs inferred radial coordinates in the four networks. Only LaBNE’s coordinates are shown, because all the methods follow the same strategy to infer them. **b** Connection probabilities as a function of hyperbolic distances measured with the coordinates inferred by each method. **c** Greedy routing efficiency when the inferred hyperbolic coordinates are used as addresses to send packets between 1000 randomly selected source-target pairs. **d** Hop stretch of successful packet deliveries for the considered source-target pairs. Red diamonds indicate the average hop stretch. **e** Time needed by each method to embed the networks to hyperbolic space

As described in the Methods, new nodes in the PS model acquire radial coordinates *r*_*t*_=2 ln*t* that depend on their birth-time *t*. This means that the probability of finding a node that is close to the centre of the hyperbolic circle containing the network, is exponentially lower than the probability to find a peripheral node (Alanis-Lobato and Andrade-Navarro 2016). When a new node is added to the system and the existing ones change their radial position according to *r*_*s*_(*t*)=*β* *r*_*s*_+(1−*β*)*r*_*t*_, with *β*=1/(*γ*−1), their seniority is attenuated by increasing their distances to every newly added node (Papadopoulos et al. 2012). Consequently, LaBNE, HyperMap and LaBNE+HM obtain radial coordinates for the *N* nodes in a network via *r*_*i*_=2*β* ln(*i*)+2(1−*β*) ln(*N*), where nodes *i*={1,2,…,*N*} are the network nodes sorted decreasingly by degree. Figure 3 a, Additional file 1: Figures S1a and S2a (*γ*={2.5,2.25,2.75} respectively) show that this is a good strategy, as inferred radial coordinates are practically the same as the ones from the input networks.

To verify whether the similarity dimension is also properly inferred, we measured pairwise hyperbolic distances between nodes, using the coordinates found by each technique, and computed the fraction of connected node pairs amongst all pairs separated by a certain distance. When distances are short, this fraction should be close to 1, when they are long it should be close to 0. Figure 3 b, Additional file 1: Figures S1b and S2b show that this is indeed the case for LaBNE+HM and HyperMap, but LaBNE’s coordinates suffer from the problems discussed in Fig. 1, especially in networks with low $\bar {c}$ (high *T*).

One of the big advantages of revealing the geometry underlying a complex network is that it enables the analysis of its navigation efficiency. An important function of complex systems is the routing of information or signals (that we refer to as packets here) without global knowledge of the network topology, avoiding loss of the packet and following short paths (Boguñá et al. 2009; Papadopoulos et al. 2010). We check if it is possible to send packets from a source node to a target one using only local topological information, i.e. the address of the source’s direct neighbours (encoded by their hyperbolic coordinates) and the address of the target. The source node ships a packet to the direct neighbour that is hyperbolically closer to the target node, the recipient neighbour does the same with its direct neighbours and so on, until the packet reaches the target. This process is known as greedy routing (Kleinberg 2007; Krioukov et al. 2010; Papadopoulos et al. 2010). If, in the delivery process, a neighbour sends the packet to the previously visited node, i.e. it falls into a loop, the packet is dropped and the delivery is flagged as unsuccessful. For each artificial network, we considered 1000 source-target pairs and measured the percentage of successfully delivered packets and the hop stretch, i.e. the length of the utilised greedy path divided by the length of the shortest path between source and target (Krioukov et al. 2010). As we can see in Fig. 3 c, Additional file 1: Figures S1c and S2c, routing efficiency is very high (close to 100 *%*, i.e. almost no packets were dropped) in heterogeneous and strongly clustered networks (low *γ* and *T*, Additional file 1: Figure S1c) and is reduced in networks with high *γ* and *T* (Additional file 1: Figure S2c). LaBNE’s performance is highly affected in the latter case (efficiency below 70 *%*), but it is the best in the former (practically 100 *%* efficiency). Coordinates inferred by LaBNE+HM and HyperMap allow for efficient navigability in practically all the analysed cases (efficiency above 80 *%*). Moreover, greedy paths are optimal for all techniques, as evidenced by the average hop stretches being close to 1, which indicates that greedy paths are also shortest paths (Fig. 3 d, Additional file 1: Figures S1d and S2d).

Finally, we recorded the amount of time required by each embedding technique to map the considered networks into $\mathbb {H}^{2}$. From Fig. 3 e, Additional file 1: Figures S1e and S2e, we can conclude that LaBNE+HM represents a very good trade-off between accuracy and embedding time.

### Performance in real networks

Given the accuracy and time performance achieved by LaBNE+HM in artificial networks, we used it to infer the hyperbolic coordinates of nodes in three real ones (see Table 1 and Methods) and repeated the analyses of the previous section. As already discussed, the width of the window used by the HyperMap part of LaBNE+HM depends on the quality of the embedding produced by LaBNE, which is better if the input network has high clustering and average node degree. Consequently, the three real networks analysed here were chosen to investigate the performance of LaBNE, HyperMap and LaBNE+HM in the low, medium and high clustering coefficient scenarios (see Table 1). Furthermore, these datasets represent complex systems from different domains: the high quality human protein interactome (PIN) models the relationships between proteins in the human cell (low $\bar {c}$, high *T*), in the Pretty-Good-Privacy network (PGP) users share encryption keys with people they trust (medium $\bar {c}$ and *T*) and the US airport network (AIR) connects cities in the US if there is a flight between them (high $\bar {c}$, low *T*). The Methods and Additional file 1: Figure S3 describe how temperatures were determined for each network, based on their actual clustering coefficients. Taking the obtained temperatures as a reference, in order to apply LaBNE+HM on the three real networks we considered windows *w*=5*π*/3 radians (300°) for the PIN, *w*=*π*/4 radians (45°) for the PGP and *w*=*π*/12 radians (15°) for the AIR (see Methods for strategies to choose *w*).

**Table 1:** The three real networks analysed in this paper: the high quality protein interaction network (PIN), the Pretty-Good-Privacy web of trust (PGP) and the US airport network (AIR)

Network	*N*	*L*	2*m*	*γ*	$\bar {c}$	*T*
PIN	10824	66154	12.22	2.66	0.18	0.77
PGP	14367	37900	5.28	2.14	0.47	0.43
AIR	500	2980	11.92	2.01	0.73	0.15

The number of nodes *N* and links *L*, average node degree 2*m*, scaling exponent *γ*, clustering coefficient $\bar {c}$ and inferred network temperature *T* are reported for each network

In the PS model, radial coordinates are directly proportional to node birth-times, i.e. if a node *i* is close to the origin of the hyperbolic circle (*r*_*i*_→0), it means that it was born early in the evolution of the complex system (Papadopoulos et al. 2012). We could not test if this is the case in the PGP or the AIR, as the considered network snapshots lack node birth-time information. However, it was possible to deduce approximate birth-times for the proteins of the PIN (see Methods). Node radial coordinates inferred by LaBNE, HyperMap and LaBNE+HM were compared to actual protein birth-times and, as shown in Additional file 1: Figure S4, nodes that are close to the centre of the hyperbolic space are older than peripheral ones. This shows that, even when the identity of the network nodes is unknown, we can have an idea of their history in the system under study, based merely on their degree and, consequently, their inferred radial positions.

**Fig. 4:**
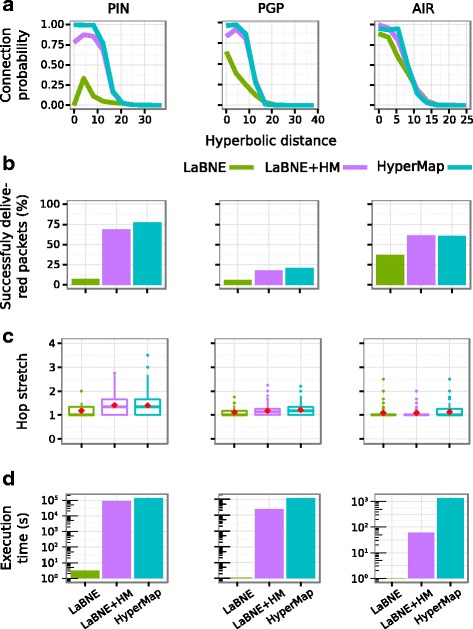
Benchmarking on real networks. **a** Connection probabilities as a function of hyperbolic distances measured with the coordinates inferred by each method. **b** Greedy routing efficiency when the inferred hyperbolic coordinates are used as addresses to send packets between 1000 randomly selected source-target pairs. **c** Hop stretch of successful packet deliveries for the considered source-target pairs. Red diamonds indicate the average hop stretch. **d** Time needed by each method to embed each real network to hyperbolic space

When it comes to connection probabilities, navigation efficiency and greedy path optimality, the results on real networks agree with what we observed in the previous section. LaBNE struggles with performing a good embedding of the PIN (low $\bar {c}$), but improves in the PGP and the AIR (medium and high $\bar {c}$). This is reflected in the low connection probability at short hyperbolic distances and the poor routing efficiency in the protein network, whereas these indicators are better in the other two systems (see Fig. 4 a-c). HyperMap, on the other hand, is quite stable in all three cases, but it required days to complete the embeddings (see Fig. 4 a-d). LaBNE+HM is in the middle of these two extremes, with much better performance than LaBNE in terms of connectivity and greedy routing and shorter execution times in general (see Fig. 4 a-d). It is also clear how the coordinates inferred by LaBNE impact LaBNE+HM’s results. In the PIN, it was necessary to probe for better angular coordinates in a very wide window, which in practical terms resulted in neglecting LaBNE’s angles and finding new ones from scratch, increasing execution time (see Fig. 4 a-d). At the other extreme, the AIR does not need much angle refinement because the configuration passed on to HyperMap is already good, which derives in a very fast yet accurate embedding (see Fig. 4 a-d).

To conclude, it is worth noting that greedy routing efficiency is quite poor in the PGP and neither HyperMap, nor LaBNE or LaBNE+HM can find hyperbolic coordinates that increase the percentage of successfully delivered packets in this network (see Fig. 4 b,c). This may be explained by the fact that the PGP is the only assortative network from the three analysed (Papadopoulos et al. 2012). In assortative networks, nodes of similar degrees tend to be connected, as opposed to disassortative networks, where high-degree nodes tend to connect with low-degree ones (Newman 2002). As shown by Krioukov and colleagues, heterogeneity is a key feature of more navigable networks, because routing paths follow a zoom-in-zoom-out hierarchical pattern (Krioukov et al. 2010). They exhibit a greedy behaviour that takes a packet from a source node towards the centre of the hyperbolic space, where high-degree nodes lie (zoom-out coarse grain search). These nodes at the top of the network hierarchy process and ship the packet back to the periphery of the hyperbolic plane, towards low-degree nodes, until the packet reaches its target (zoom-in fine grain search) (Boguñá et al. 2009; Cannistraci et al. 1613; Krioukov et al. 2010). In an assortative network, this hierarchy is less clear and a packet may get stuck on its way to the target, reducing routing efficiency.

Interestingly, in the AIR, with all the characteristics of a navigable network (high heterogeneity, clustering and disassortativity), routing efficiency only reaches 61 *%* (see Fig. 4 b). Since this network is comprised of only the 500 busiest airports in the US, from a total of 19512 airport facilities listed by the Federal Aviation Administration (http://www.faa.gov/), the over-representation of hub airports can lead to packets being unable to leave them and reach their less-connected peripheral targets.

## Conclusions

Scale-invariance, self-similarity and strong clustering, properties present in complex systems and geometric objects alike, have led to the proposal that the network representations of the former lie on a geometric space, where distance constraints play important roles in the formation of links between system components (Boguñá et al. 2009; Cannistraci et al. 2013b; Krioukov et al. 2010; Papadopoulos et al. 2012). Our results and those of others support the idea that hyperbolic space is a good candidate to host complex networks, as it allows for the precise description of their formation and function (Alanis-Lobato et al. 2016; Krioukov et al. 2010; Papadopoulos et al. 2012; Papadopoulos F et al. 2015b; Papadopoulos et al. 2015a).

In consequence, efficient methods to embed networks into this space are needed. In this article we exploit the strengths of two such methods, LaBNE and HyperMap, to quickly obtain accurate embeddings of artificial and real networks. Although it is difficult to validate this claim in the latter case, we have tested the performance of these embedding techniques from a node birth-time, connectivity and navigability perspective. Furthermore, we have shown that good embeddings to $\mathbb {H}^{2}$ are possible in a short amount of time, especially in heterogeneous, dense and strongly clustered networks. Our work also highlights the strengths and limitations of LaBNE and HyperMap, and their impact on LaBNE+HM, the proposed hybrid approach that takes LaBNE’s embeddings and refines them with HyperMap.

It should be noted that techniques for embedding networks to generic low-dimensional spaces have been proposed to facilitate their visualisation and analysis (Belkin and Niyogi 2001; Cannistraci et al. 2013b; Cayton 2005; Kuchaiev et al. 2009; Newman and Peixoto 2015; Tenenbaum 2000; You et al. 2010; Zemel and Carreira-Perpiñán 2004). Nevertheless, LaBNE, HyperMap and LaBNE+HM deal specifically with the embedding to the two-dimensional hyperbolic plane. As our results suggest, this space provides an accurate reflection of the geometry of real networks (Alanis-Lobato et al. 2016; Krioukov et al. 2010; Papadopoulos et al. 2012) and facilitates their visual inspection and analysis. This prompts us to further improve existing hyperbolic mapping techniques, as massive networks with billions of nodes become more and more common in the age of Big Data.

## Methods

### The PS model

The PS model (Papadopoulos et al. 2012) on the hyperbolic plane of curvature *K*=−1 is formulated as follows: (1) initially the network is empty; (2) at time *t*≥1, a new node *t* appears at coordinates (*r*_*t*_,*θ*_*t*_) with *r*_*t*_=2 ln*t* and *θ*_*t*_ uniformly distributed on [ 0,2*π*], and every existing node *s*<*t* increases its radial coordinate according to *r*_*s*_(*t*)=*β* *r*_*s*_+(1−*β*)*r*_*t*_ with *β*=1/(*γ*−1)∈[ 0,1]; (3) new node *t* picks a randomly chosen node *s*<*t* that is not already connected to it and links with it with probability $p(x_{st}) = 1 / \left [1 + e^{(x_{st} - R_{t})/2T}\right ]$, where parameter *T*, the network temperature, controls the network’s clustering coefficient, $R_{t} = r_{t} - 2\ln \left [\frac {2T(1 - e^{-(1 - \beta)r_{t}/2})}{m(1 - \beta)\sin (\pi T)}\right ]$ is the current radius of the hyperbolic circle containing the network, *x*_*st*_=*r*_*s*_+*r*_*t*_+2 ln(*θ*_*st*_/2) is the hyperbolic distance between nodes *s* and *t* and *θ*_*st*_ is the angle between the nodes; (4) repeat step 3 until node *t* gets connected to *m* different nodes; (5) repeat steps 1-4 until the network is comprised of *N* nodes. Note that if *T*→0, $R_{t} = r_{t} - 2\ln \left [\frac {2(1 - e^{-(1 - \beta)r_{t}/2})}{\pi m(1 - \beta)}\right ]$. In addition, if *β*=1, existing nodes do not change their radial coordinates and $R_{t} = r_{t} - 2\ln \left (\frac {T r_{t}}{m\sin (\pi T)}\right)$.

### HyperMap

HyperMap (Papadopoulos F et al. 2015b) is a Maximum Likelihood Estimation method to embed a network to hyperbolic space. It finds node coordinates by replaying the network’s hyperbolic growth and, at each step, maximising the likelihood that it was produced by the PS model (Papadopoulos F et al. 2015b). For embedding to the hyperbolic plane of curvature *K*=−1 it works as follows: (1) nodes are sorted decreasingly by degree and labelled *i*={1,2,…,*N*} from the top of the sorted list; (2) node *i*=1 is born and assigned radial coordinate *r*_1_=0 and a random angular coordinate *θ*_1_∈[ 0,2*π*]; (3) for each node *i*={2,3,…,*N*}: (3.1) node *i* is born and assigned radial coordinate *r*_*i*_=2 ln*i*; (3.2) the radial coordinate of every existing node *j*<*i* is increased according to *r*_*j*_(*i*)=*β* *r*_*j*_+(1−*β*)*r*_*i*_; (3.3) node *i* is assigned the angular coordinate *θ*_*i*_ maximising the likelihood $\mathcal {L}^{i}_{L} = \prod _{1 \leq j < i} p(x_{ij})^{\alpha _{ij}}(1 - p(x_{ij}))^{1 - \alpha _{ij}}$. *β* and *p*(*x*_*ij*_) are defined as in the PS model and *α*_*ij*_ is 1 if nodes *i* and *j* are connected and 0 otherwise. The maximisation of $\mathcal {L}^{i}_{L}$ is performed numerically by trying different values of *θ* in [ 0,2*π*], separated by intervals *Δ* *θ*=1/*i*, and then choosing the one that produces the greatest $\mathcal {L}^{i}_{L}$.

Since the angular coordinates yielded by this link-based likelihood are not very accurate for small *i* (i.e. for high degree nodes) (Papadopoulos F et al. 2015b), the fast version of HyperMap used in this paper uses information on the final number of common neighbours between these old nodes via the maximisation of the log-likelihood $\ln \mathcal {L}^{i}_{CN} = (i - 1)\ln \frac {1}{\sqrt {2\pi }} - \sum _{j=1}^{i-1}\ln \sigma (i,j,\theta _{i}, \theta _{j}) - \sum _{j=1}^{i-1}\frac {n_{ij}^{t} - \mu (i,j,\theta _{i}, \theta _{j})}{2\sigma ^{2}(i,j,\theta _{i}, \theta _{j})}$, where *μ* is the mean number of common neighbours *n*_*ij*_ between *i* and *j* and *σ* ^2^ is the associated variance (Papadopoulos et al. 2015a). This hybrid version of HyperMap is *O*(*N* ^3^) and to speed it up, Papadopoulos and colleagues resort to the following heuristic: for nodes *i* with degree *k*_*i*_<*k*_*speedup*_, an initial estimate $\theta _{i}^{init}$ of their angular coordinate is computed by considering only the previous nodes *j*<*i* that are their neighbours; these estimates are then refined, searching for the final *θ*_*i*_ within a small region around $\theta _{i}^{init}$. The fast hybrid version of HyperMap with *k*_*speedup*_=10 is the one used throughout this work and is the one that refines LaBNE’s embeddings in LaBNE+HM. We refer the reader to (Papadopoulos et al. 2015a) for more details on the speed-up heuristic and the derivation of $\mathcal {L}^{i}_{CN}$. Finally, even when correction steps can be used together with the fast hybrid HyperMap, their effect on this method has been reported not to be significant (Papadopoulos et al. 2015a) and they are not considered here.

### LaBNE

Let us consider only undirected, unweighted, single-component networks, as LaBNE is only applicable to such networks (Alanis-Lobato et al. 2016; Belkin and Niyogi 2001). Moreover, let us assume that these networks are scale-free (with scaling exponent *γ*∈[ 2,3]) and have a clustering coefficient $\bar {c}$ that is significantly bigger than expected by chance. These networks are graphs *G*=(*V*,*E*) with *N*=|*V*| nodes and *L*=|*E*| edges connecting them. An undirected, unweighted graph can be represented by an *N*×*N* adjacency matrix *A*_*i*,*j*_=*A*_*j*,*i*_ ∀*i*,*j*, whose entries are 1 if there is an edge between nodes *i* and *j* and 0 otherwise. The graph Laplacian is a transformation of *A* given by *L*=*D*−*A*, where *D* is a matrix with the node degrees on its diagonal and 0 elsewhere.

The Laplacian-based embedding of a complex network to the two-dimensional hyperbolic plane $\mathbb {H}^{2}$, represented by the interior of a Euclidean circle (Krioukov et al. 2010), is given by the *N*×2 matrix *Y*=[*y*_1_,*y*_2_] where the *i*th row, *Y*_*i*_, provides the embedding coordinates of node *i*. This corresponds to minimising $\frac {1}{2} \sum _{i,j} A_{i, j}||Y_{i} - Y_{j}||^{2} = \text {tr}(Y^{T}LY)$, which reduces to $\phantom {\dot {i}\!}Y_{emb} = \text {min}_{Y^{T}DY = I}\text {tr}(Y^{T}LY)$ with *D* as defined above, *I* the identity matrix, *M* ^*T*^ the transpose of *M* and tr(*M*) the trace of *M*. Finally, *Y*_*emb*_, the matrix that minimises this objective function, is formed by the two eigenvectors with smallest non-zero eigenvalues that solve the generalised eigenvalue problem *L* *Y*=*λ* *D* *Y* (see (Alanis-Lobato et al. 2016) for a detailed derivation of this result).

To complete the mapping to $\mathbb {H}^{2}$, angular node coordinates are obtained via **θ**=arctan(*y*_2_/*y*_1_) and radial coordinates are chosen so as to resemble the rank of each node according to its degree. This is achieved via *r*_*i*_=2*β* ln(*i*)+2(1−*β*) ln(*N*), where nodes *i*={1,2,…,*N*} are the network nodes sorted decreasingly by degree and *β*=1/(*γ*−1) (Krioukov et al. 2010; Papadopoulos et al. 2012). Finally, to further refine the embedding, angular coordinates are re-adjusted by spreading them uniformly in [ 0,2*π*], based on the order of the angles inferred initially.

The strategy followed by LaBNE is valid, because the native representation of $\mathbb {H}^{2}$, in which the hyperbolic space is contained in a Euclidean disc and Euclidean and hyperbolic distances from the origin are equivalent, is a conformal model. This means that Euclidean angular separations between nodes are equivalent to hyperbolic ones (Krioukov et al. 2010). On the other hand, the radial arrangement of nodes corresponds to a quasi-uniform distribution of radial coordinates in the disc (Krioukov et al. 2010; Alanis-Lobato and Andrade-Navarro 2016).

### Network datasets

For the three network datasets used in this paper, self-loops and multiple edges were discarded and only the largest connected component was considered.

The high-quality protein interaction network (PIN) is a stringent subset of the Human Integrated Protein-Protein Interaction rEference (HIPPIE) (Schaefer et al. 2012; Alanis-Lobato et al. 2016). HIPPIE retrieves interactions between human proteins from major expert-curated databases and calculates a score for each one, reflecting its combined experimental evidence. This score is a function of the number of studies supporting the interaction, the quality of the experimental techniques used to measure it and the number of organisms in which the orthologs of the interacting human proteins interact as well. In this paper, only interactions with confidence scores ≥0.72 (the upper quartile of all scores) in release 2.0 were considered. The raw version of this network is available at http://cbdm-01.zdv.uni-mainz.de/~mschaefer/hippie/download.php. To determine the birth-time of the PIN nodes, proteins from the manually curated database SwissProt were clustered based on near full-length similarity and/or high threshold of sequence identity using FastaHerder2 (Mier and Andrade-Navarro 2016). If proteins from two evolutionarily distant organisms are present in one cluster, this suggests that the protein family is ancient. The minimum common taxonomy from all proteins that are part of a cluster was taken as an indication of the cluster’s age. Each node of the PIN was assigned to one of the following age clusters: Cellular organisms, Metazoa, Chordata, Mammalia, Euarchontoglires or Primates.

Pretty-Good-Privacy (PGP) is a data encryption and decryption program for secure data communication. In a PGP web of trust, each user (node) knows the public key of a group of people he trusts. When user A wants so send information to user B, this information is encrypted with B’s public key and signed with A’s private key. When B receives the information, he verifies that the message is coming from one of the users he trusts and decrypts it with his private key (Schneier 1996). This encryption and decryption event, forms a directed link between users A and B. In this article, however, the edge directionality of this network is not considered. This is not a problem for the interpretation of the network if we assume that by sharing a key, two users reciprocally endorse their trust in each other (Papadopoulos et al. 2012). From the four temporal snapshots of the undirected PGP network collected by Jörgen Cederlöf (Cederlöf 2003), only the one corresponding to the period between April and October 2003 was used here. The raw PGP data is available at http://www.lysator.liu.se/~jc/wotsap/wots2/.

The airport network (AIR) corresponds to the connections between the 500 busiest commercial airports in the United States. Two airports are linked if there was a flight scheduled between them in 2002. This dataset was used in (Colizza et al. 2007) and is available at http://opsahl.co.uk/tnet/datasets/USairport500.txt or https://sites.google.com/site/cxnets/US_largest500_airportnetwork.txt.

### Real network temperature determination

To determine an appropriate temperature for the three real networks used in this work, we take advantage of results showing that clustering decreases almost linearly with network temperature, until it is 0 for *T*=1 (Krioukov et al. 2010; Papadopoulos et al. 2012). For each real network, ten artificial networks, with the same structural properties as the real system at hand, are generated with the PS model using *T*=0. The clustering coefficient of the ten networks is averaged and used as y-intercept, while the point $(T = 1, \bar {c} = 0)$ is used as x-intercept. We can then determine the equation of this line and use it to compute a *T* for each real network, based on its clustering coefficient (see Table 1 and Additional file 1: Figure S3).

### Choice of window size in LaBNE+HM

The windows *w* used in the artificial and real networks analysed in this paper were chosen based on their clustering coefficients, determined temperatures and the performance of LaBNE when applied to them. Although we consider the latter as a very good point of reference to decide on window widths, given the speed of LaBNE, a more automated and fast strategy would be to consider a linear or quadratic relationship between *w* and temperature *T*∈[ 0,1], *w*=2*π* *T* or *w*=2*π* *T* ^2^ for example. Note that this might result in windows wider than needed and slower LaBNE+HM embedding times, but it would produce very good and refined mappings in most cases.

### Hardware used for experiments

All the experiments presented in this paper were executed on a Lenovo ThinkPad 64-bit with 7.7 GB of RAM and an Intel Core i7-4600U CPU @ 2.10 GHz × 4, running Ubuntu 16.04 LTS. The only exceptions were the packet delivery and the connection probability experiments, which were executed on nodes with 30 GB of RAM, within the Mogon computer cluster at the Johannes Gutenberg Universität in Mainz.

## Additional file

Additional file 1 Supplementary information. (PDF 539 kb)
