# Supplementary material for: Manifold learning and maximum likelihood estimation for hyperbolic network embedding
Source: Appl Netw Sci. 2016 Nov 15;1(1):10. doi: 10.1007/s41109-016-0013-0 (PMC6245200; doi:10.1007/s41109-016-0013-0)
Supplement: Supplementary file 1 — Supplementary information. (PDF 539 kb) [file 41109_2016_13_MOESM1_ESM.pdf]

SUPPLEMENTARY INFORMATION

Manifold learning and maximum likelihood estimation for hyperbolic network embedding

Gregorio Alanis-Lobato[*,1,2], Pablo Mier[1,2], and Miguel A. Andrade-Navarro[1,2]

[1] *Institute of Molecular Biology, Ackermannweg 4, 55128 Mainz, Germany*
[2] *Faculty of Biology, Johannes Gutenberg Universität, Gresemundweg 2, 55128 Mainz, Germany*

[*] Corresponding author: galanisl@uni-mainz.de

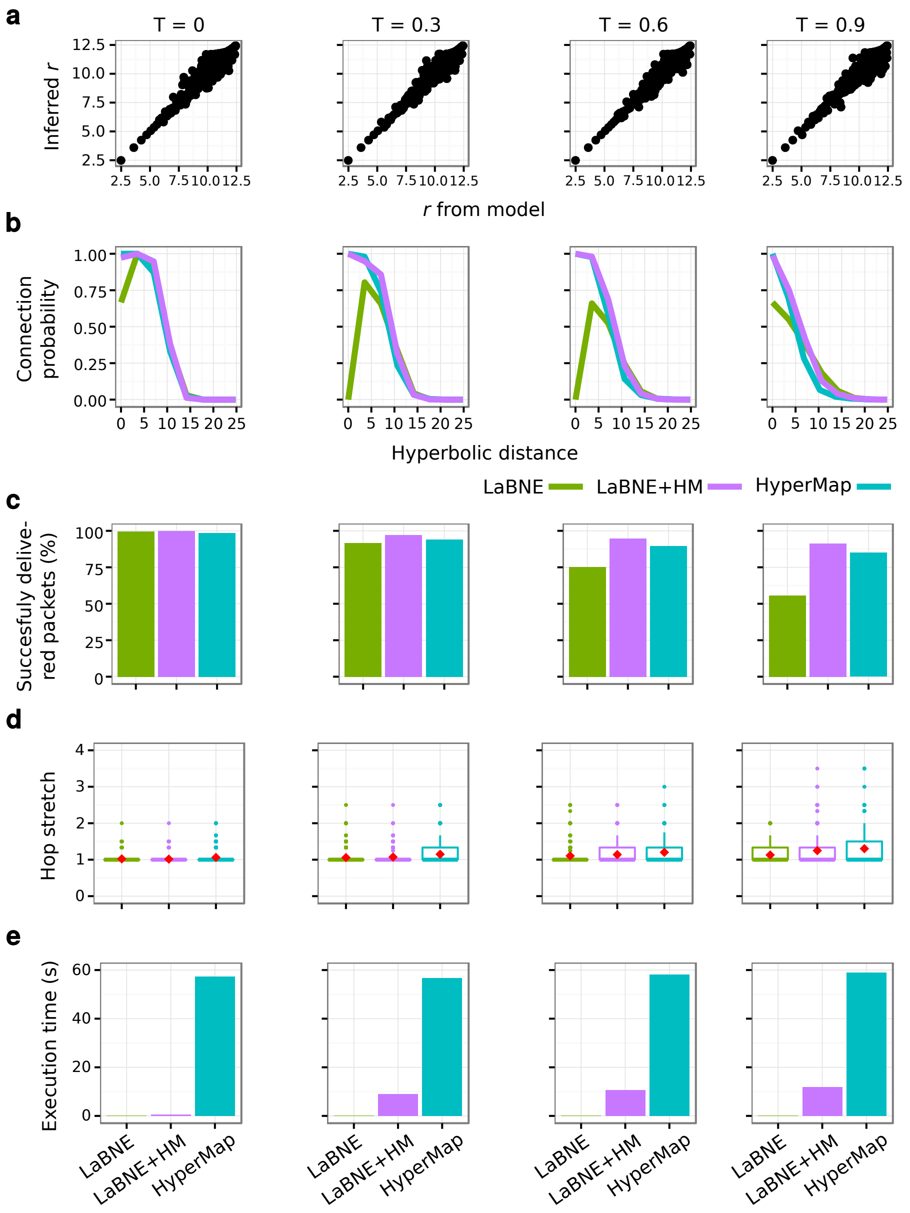

Figure S1: **Benchmarking on artificial networks** ($\gamma = 2.25$). Artificial networks with $N = 500$ nodes, average node degree $2m = 10$, scaling exponent $\gamma = 2.25$ and temperature $T = \{0, 0.3, 0.6, 0.9\}$ were embedded to hyperbolic space with LaBNE, HyperMap and LaBNE+HM. **(a)** Real vs inferred radial coordinates in the four networks. Only LaBNE's coordinates are shown because all the methods follow the same strategy to infer them. **(b)** Connection probabilities as a function of hyperbolic distances measured with the coordinates inferred by each method. **(c)** Greedy routing efficiency when the inferred hyperbolic coordinates are used as addresses to send packets between 1000 randomly selected source-target pairs. **(d)** Hop stretch of successful packet deliveries for the considered source-target pairs. Red diamonds indicate the average hop stretch. **(e)** Time needed by each method to embed the networks to hyperbolic space.

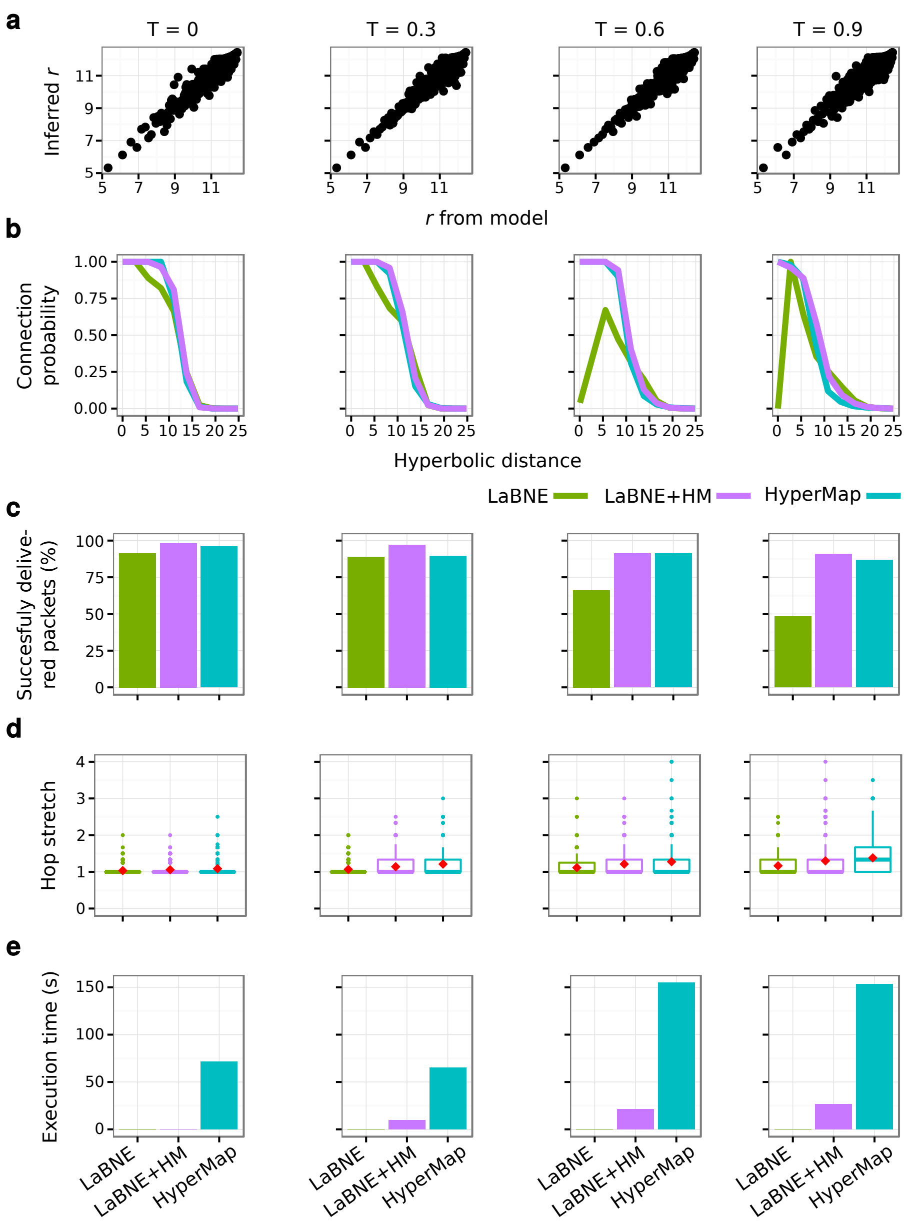

Figure S2: **Benchmarking on artificial networks** ($\gamma = 2.75$)**.** Artificial networks with $N = 500$ nodes, average node degree $2m = 10$, scaling exponent $\gamma = 2.75$ and temperature $T = \{0, 0.3, 0.6, 0.9\}$ were embedded to hyperbolic space with LaBNE, HyperMap and LaBNE+HM. **(a)** Real vs inferred radial coordinates in the four networks. Only LaBNE's coordinates are shown because all the methods follow the same strategy to infer them. **(b)** Connection probabilities as a function of hyperbolic distances measured with the coordinates inferred by each method. **(c)** Greedy routing efficiency when the inferred hyperbolic coordinates are used as addresses to send packets between 1000 randomly selected source-target pairs. **(d)** Hop stretch of successful packet deliveries for the considered source-target pairs. Red diamonds indicate the average hop stretch. **(e)** Time needed by each method to embed the networks to hyperbolic space.

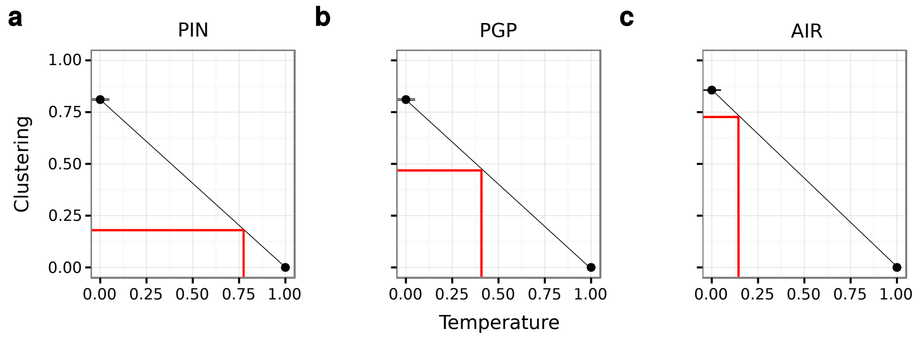

Figure S3: **Network temperature determination.** Strategy used to determine an appropriate temperature for the three real networks used in this work: **(a)** the high-quality human protein interactome (PIN), **(b)** the Pretty-Good-Privacy web of trust (PGP) and **(c)** the US airport network (AIR). In each case, ten artificial networks, with the same structural properties as the real system at hand, are generated with the PS model using $T = 0$. Since clustering has been show to decrease almost linearly with temperature until it is 0 for $T = 1$, the clustering of the ten networks is averaged and used as y-intercept, while the point $(T = 1, \bar{c} = 0)$ is used as x-intercept. We can then find the equation of this line and determine the real network's temperature using its clustering coefficient as shown in the figures with red lines.

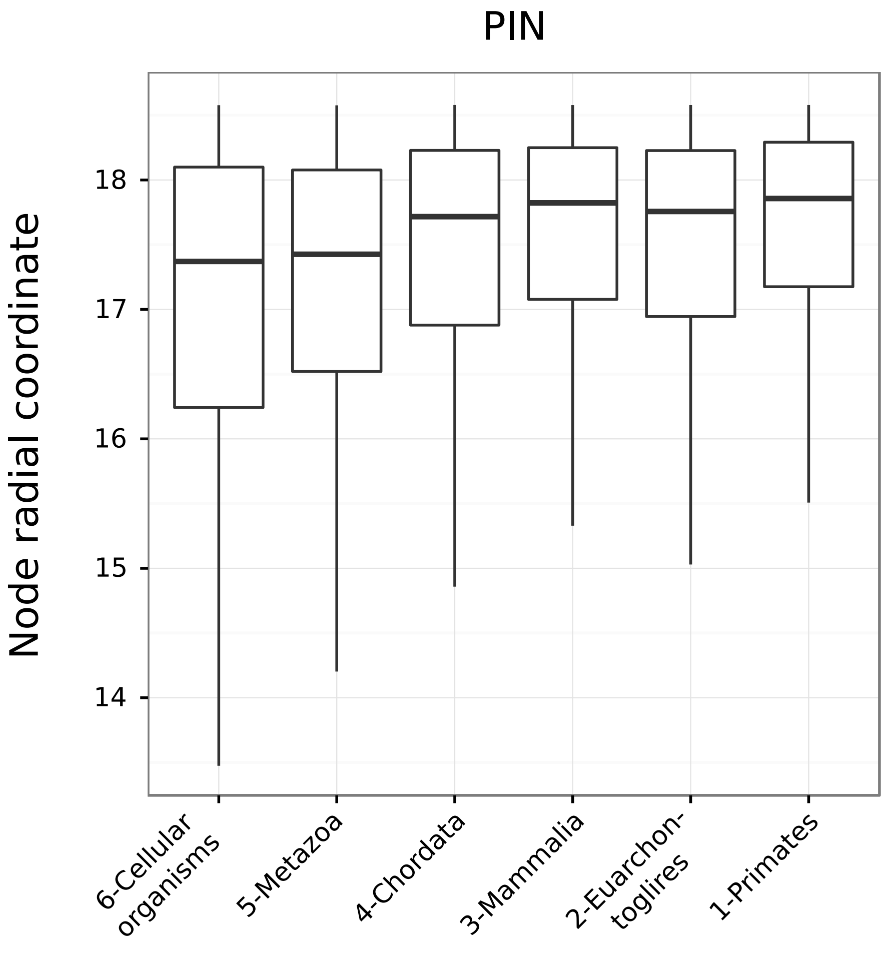

Figure S4: **Inferred radial coordinates resemble actual protein birth-times.** Old proteins in the analysed human protein interactome, i.e. proteins present in all cellular organisms, have radial coordinates that are close to the centre of the hyperbolic disc containing the network, as opposed to younger, species-specific proteins, which are placed in the circle's periphery. Only LaBNE's coordinates are shown, because all the methods follow the same strategy to infer them.
